# [μ-1,4-Bis(1,2,4-triazol-1-ylmeth­yl)benzene]­bis­[aqua­(pyridine-2,6-dicarboxyl­ato)copper(II)] monohydrate

**DOI:** 10.1107/S1600536811022756

**Published:** 2011-06-18

**Authors:** Gui-Ying Dong, Cui-Hong He, Liu Tong-Fei, Xiao-Chen Deng, Xiao-Ge Shi

**Affiliations:** a College of Chemical Engineering, Hebei United University, Tangshan 063009, People’s Republic of China; b Qian’an College, Hebei United University, Tangshan 063009, People’s Republic of China

## Abstract

The title compound, [Cu_2_(C_7_H_3_NO_4_)_2_(C_12_H_12_N_6_)(H_2_O)_2_]·H_2_O, displays a discrete dinuclear structure, in which the central Cu^II^ atom is five-coordinated in a distorted square-based pyramidal coordination geometry and the flexible ligand 1,4-bis­(1,2,4-triazol-1-ylmeth­yl)benzene adopts a bis-monodentate bridging mode linking the Cu^II^ atoms. It is further assembled by O—H⋯O hydrogen-bond inter­actions involving both the coordinated and uncoordinated water molecules. The latter exhibits half-occupancy.

## Related literature

For the versatile conformations of the flexible 1,4-bis­(1,2,4- triazol-1-yl-meth­yl)benzene ligand and related complexes, see: Arion *et al.* (2003 ▶); Peng *et al.* (2004 ▶, 2006 ▶); Meng *et al.* (2004 ▶); Li *et al.* (2005 ▶); Lin & Dong (2007 ▶); Ding *et al.* (2009 ▶).
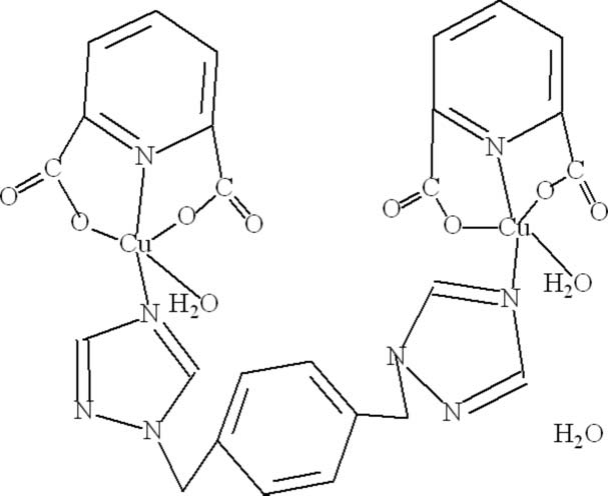


## Experimental

### 

#### Crystal data

[Cu_2_(C_7_H_3_NO_4_)_2_(C_12_H_12_N_6_)(H_2_O)_2_]·H_2_O
*M* *_r_* = 751.63
Monoclinic, 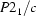

*a* = 4.9017 (4) Å
*b* = 10.3022 (9) Å
*c* = 30.178 (3) Å
β = 93.541 (1)°
*V* = 1521.0 (2) Å^3^
*Z* = 2
Mo *K*α radiation
μ = 1.47 mm^−1^
*T* = 298 K
0.20 × 0.15 × 0.11 mm

#### Data collection

Bruker SMART CCD area-detector diffractometer
Absorption correction: multi-scan (*SADABS*; Sheldrick, 1996 ▶) *T*_min_ = 0.881, *T*_max_ = 0.901
7340 measured reflections
2678 independent reflections
2256 reflections with *I* > 2σ(*I*)
*R*_int_ = 0.029

#### Refinement

*R*[*F* ^2^ > 2σ(*F* ^2^)] = 0.047
*wR*(*F* ^2^) = 0.161
*S* = 1.06
2678 reflections
217 parameters
H-atom parameters constrained
Δρ_max_ = 1.40 e Å^−3^
Δρ_min_ = −0.40 e Å^−3^

### 

Data collection: *SMART* (Bruker, 1998 ▶); cell refinement: *SAINT* (Bruker, 1998 ▶); data reduction: *SAINT*; program(s) used to solve structure: *SHELXS97* (Sheldrick, 2008 ▶); program(s) used to refine structure: *SHELXL97* (Sheldrick, 2008 ▶); molecular graphics: *SHELXTL* (Sheldrick, 2008 ▶); software used to prepare material for publication: *SHELXTL*.

## Supplementary Material

Crystal structure: contains datablock(s) I, global. DOI: 10.1107/S1600536811022756/jh2295sup1.cif

Structure factors: contains datablock(s) I. DOI: 10.1107/S1600536811022756/jh2295Isup2.hkl

Additional supplementary materials:  crystallographic information; 3D view; checkCIF report

## Figures and Tables

**Table 1:** Hydrogen-bond geometry (Å, °)

*D*—H⋯*A*	*D*—H	H⋯*A*	*D*⋯*A*	*D*—H⋯*A*
O2*W*—H2*WA*⋯O2*W*^i^	0.85	2.15	2.920 (5)	151
O2*W*—H2*WB*⋯O2*W*^ii^	0.85	1.96	2.807 (5)	179
O1*W*—H1*WA*⋯O4^iii^	0.83	1.93	2.746 (5)	168
O1*W*—H1*WB*⋯O3^iv^	0.86	1.86	2.692 (5)	164

Symmetry codes: (i) 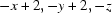; (ii) 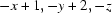; (iii) 
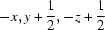
; (iv) 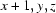.

**Fig. 1.**
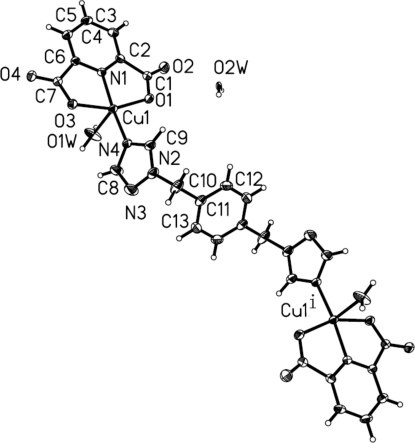
Fig. 1. The part molecular structure of (I), showing displacement ellipsoids at the 30% probability level for atoms [symmetry code: (i) -x, -y + 3, -z)

## supplementary crystallographic information

### Comment

1,4-bis(1,2,4-triazol-1-ylmethyl)benzene (btx=1,4-bis(1,2,4-triazol-1-ylmethyl)benzene) is a ditriazole-containing bridge ligand, in which the flexible nature of spacers allows the ligands to bend and rotate when coordinating to metal centers so as to conform the coordination geometries of metal ions.(Arion, *et al.*, 2003; Peng, *et al.*, 2004, 2006; Meng, *et al.*, 2004; Li *et al.*, 2005;Lin *et al.* 2007; Ding, *et al.* 2009) To further understand the coordination behavior of this ligand, we report herein the crystal structure of the title compound,(I).

The asymmetric unit of (I) contains one copper^II^,one 2,6-pyridinedicarboxylato, one half btx ligand, one coordination water molecule and one half free water molecule. The copper center is five-coordinated in distorted square-based pyramidal coordination geometry. As show in Fig.1, selected geometric parameters see table 1.Each copper^II^ is coordinated by one tridentate dipicolinato ligands *via* their carboxylate and nitrogen donors.(Cu1—N1= 1.908 (3); Cu1—O1=2.007 (3); Cu(1)—O(3)= 2.048 (3) Å) another one (Cu1—N4=1.951 (3) Å) from btx ligand together with one water molecule (Cu1—O1W = 2.217 (4) Å).Two carboxylate oxygen atoms and two nitrogen atoms define a quadrangle equatorial plane, and the water oxygen atom occupies the apical position. Each btx ligand bridges two copper atoms related by a twofold axis into dinuclear structure. The dihedral angle between the imidazole and phenyl rings is 70.0 (4)° in same btx ligang. It is noteworthy that there exist strong hydrogen-bonding interaction(table 2) involving the carboxy group oxygen atoms of dipicolinato ligands as well as coordinated and free water molecules,this may further stabilize the crytal structure.

### Experimental

A mixture of Cu(NO_3_)_2_ 3H_2_O(120.5 mg, 0.5 mmol), 2,6-Pyridinedicarboxylic acid (167 mg, 1 mmol),NaOH(80 mg, 2 mm mol), btx (60 mg, 0.5 mmol) and water (12 ml) was sealed in a 25 ml teflon-lined stainless steel reactor and heated to 413 K for 72 h. The reaction was cooled to room temperature over a period of 24 h. Blue prism crystals of 1 suitable for X-ray difraction analysis were obtained with a yield of 37%(based btx)

### Refinement

H atoms were placed in calculated positions, with C—H = 0.93 or 0.97 Å included in the final cycles of refinement using a riding model, with *U*_iso_(H) = 1.2*U*_eq_(parent atom).Water H atoms were located in Fourier difference maps and isotropically.

### Crystal data

**Table:**

[Cu_2_(C_7_H_3_NO_4_)_2_(C_12_H_12_N_6_)(H_2_O)_2_]·H_2_O	*F*(000) = 764
*M**_r_* = 751.63	*D*_x_ = 1.641 Mg m^−^^3^
Monoclinic, *P*2_1_/*c*	Mo *K*α radiation, λ = 0.71073 Å
Hall symbol: -P 2ybc	Cell parameters from 3568 reflections
*a* = 4.9017 (4) Å	θ = 22.3–3.6°
*b* = 10.3022 (9) Å	µ = 1.47 mm^−^^1^
*c* = 30.178 (3) Å	*T* = 298 K
β = 93.541 (1)°	Prism, blue
*V* = 1521.0 (2) Å^3^	0.20 × 0.15 × 0.11 mm
*Z* = 2	

### Data collection

**Table:**

Bruker SMART CCD area-detector diffractometer	2678 independent reflections
Radiation source: fine–focus sealed tube	2256 reflections with *I* > 2σ(*I*)
graphite	*R*_int_ = 0.029
φ and ω scans	θ_max_ = 25.0°, θ_min_ = 2.1°
Absorption correction: multi-scan (*SADABS*; Sheldrick, 1996)	*h* = −5→5
*T*_min_ = 0.881, *T*_max_ = 0.901	*k* = −12→12
7340 measured reflections	*l* = −35→27

### Refinement

**Table:**

Refinement on *F*^2^	Primary atom site location: structure-invariant direct methods
Least-squares matrix: full	Secondary atom site location: difference Fourier map
*R*[*F*^2^ > 2σ(*F*^2^)] = 0.047	Hydrogen site location: inferred from neighbouring sites
*wR*(*F*^2^) = 0.161	H-atom parameters constrained
*S* = 1.06	*w* = 1/[σ^2^(*F*_o_^2^) + (0.1045*P*)^2^ + 1.8845*P*] where *P* = (*F*_o_^2^ + 2*F*_c_^2^)/3
2678 reflections	(Δ/σ)_max_ < 0.001
217 parameters	Δρ_max_ = 1.40 e Å^−^^3^
0 restraints	Δρ_min_ = −0.40 e Å^−^^3^

### Special details

**Table:**

Geometry. All e.s.d.'s (except the e.s.d. in the dihedral angle between two l.s. planes) are estimated using the full covariance matrix. The cell e.s.d.'s are taken into account individually in the estimation of e.s.d.'s in distances, angles and torsion angles; correlations between e.s.d.'s in cell parameters are only used when they are defined by crystal symmetry. An approximate (isotropic) treatment of cell e.s.d.'s is used for estimating e.s.d.'s involving l.s. planes.
Refinement. Refinement of *F*^2^ against ALL reflections. The weighted *R*-factor *wR* and goodness of fit *S* are based on *F*^2^, conventional *R*-factors *R* are based on *F*, with *F* set to zero for negative *F*^2^. The threshold expression of *F*^2^ > σ(*F*^2^) is used only for calculating *R*-factors(gt) *etc*. and is not relevant to the choice of reflections for refinement. *R*-factors based on *F*^2^ are statistically about twice as large as those based on *F*, and *R*- factors based on ALL data will be even larger.

### Fractional atomic coordinates and isotropic or equivalent isotropic displacement parameters (Å^2^)

**Table:**

	*x*	*y*	*z*	*U*_iso_*/*U*_eq_	Occ. (<1)
Cu1	0.08344 (10)	0.98034 (5)	0.149509 (16)	0.0318 (2)	
O3	−0.1809 (6)	0.9403 (3)	0.19769 (10)	0.0376 (7)	
O1	0.3528 (6)	0.9508 (3)	0.10311 (10)	0.0392 (7)	
N1	0.1905 (7)	0.8071 (3)	0.16460 (11)	0.0300 (8)	
C7	−0.1409 (9)	0.8318 (4)	0.21771 (14)	0.0328 (9)	
O4	−0.2555 (7)	0.7960 (3)	0.25024 (11)	0.0469 (8)	
N4	−0.0779 (7)	1.1422 (3)	0.12697 (12)	0.0346 (8)	
C2	0.3801 (9)	0.7519 (4)	0.14164 (14)	0.0335 (9)	
C3	0.4592 (10)	0.6261 (4)	0.15103 (16)	0.0419 (11)	
H3	0.5917	0.5859	0.1350	0.050*	
C6	0.0697 (8)	0.7462 (4)	0.19736 (13)	0.0309 (9)	
O2	0.6818 (8)	0.8113 (4)	0.08671 (12)	0.0565 (10)	
C11	−0.1952 (9)	1.4310 (5)	0.02197 (15)	0.0415 (11)	
C1	0.4833 (9)	0.8436 (4)	0.10695 (14)	0.0358 (10)	
C5	0.1422 (10)	0.6216 (4)	0.20832 (16)	0.0417 (11)	
H5	0.0613	0.5787	0.2312	0.050*	
C12	−0.0882 (11)	1.3819 (5)	−0.01557 (17)	0.0486 (12)	
H12	−0.1473	1.3016	−0.0266	0.058*	
C4	0.3384 (10)	0.5605 (5)	0.18465 (17)	0.0453 (11)	
H4	0.3890	0.4754	0.1914	0.054*	
O1W	0.3552 (8)	1.0793 (4)	0.20033 (14)	0.0698 (13)	
N2	−0.2780 (8)	1.2853 (4)	0.08451 (13)	0.0439 (10)	
C13	−0.1072 (11)	1.5492 (5)	0.03729 (17)	0.0492 (12)	
H13	−0.1788	1.5841	0.0625	0.059*	
C10	−0.4037 (11)	1.3552 (6)	0.04620 (19)	0.0576 (15)	
H10A	−0.5411	1.4144	0.0562	0.069*	
H10B	−0.4942	1.2936	0.0259	0.069*	
C9	−0.1903 (11)	1.1643 (5)	0.08746 (16)	0.0465 (12)	
H9	−0.2064	1.1037	0.0646	0.056*	
N3	−0.2233 (16)	1.3460 (5)	0.12323 (17)	0.092 (2)	
C8	−0.1028 (17)	1.2542 (6)	0.14744 (19)	0.080 (2)	
H8	−0.0398	1.2672	0.1768	0.096*	
O2W	0.746 (3)	0.9295 (7)	0.0049 (2)	0.092 (4)	0.50
H2WA	0.9152	0.9472	0.0077	0.137*	0.50
H2WB	0.5966	0.9714	0.0018	0.137*	0.50
H1WA	0.3201	1.1370	0.2183	0.137*	
H1WB	0.5177	1.0475	0.2023	0.137*	

### Atomic displacement parameters (Å^2^)

**Table:**

	*U*^11^	*U*^22^	*U*^33^	*U*^12^	*U*^13^	*U*^23^
Cu1	0.0373 (4)	0.0240 (3)	0.0348 (4)	0.0079 (2)	0.0068 (2)	0.00618 (19)
O3	0.0425 (17)	0.0334 (16)	0.0376 (16)	0.0129 (14)	0.0083 (13)	0.0073 (14)
O1	0.0469 (18)	0.0330 (16)	0.0390 (17)	0.0053 (14)	0.0130 (14)	0.0077 (13)
N1	0.0351 (19)	0.0255 (17)	0.0296 (18)	0.0040 (14)	0.0026 (14)	0.0005 (14)
C7	0.037 (2)	0.030 (2)	0.033 (2)	0.0001 (18)	0.0042 (18)	0.0012 (17)
O4	0.063 (2)	0.0381 (18)	0.0418 (18)	0.0049 (16)	0.0205 (16)	0.0073 (15)
N4	0.042 (2)	0.0274 (18)	0.035 (2)	0.0085 (15)	0.0039 (15)	0.0055 (15)
C2	0.038 (2)	0.030 (2)	0.032 (2)	0.0025 (18)	0.0025 (17)	−0.0045 (17)
C3	0.047 (3)	0.031 (2)	0.048 (3)	0.013 (2)	0.009 (2)	−0.003 (2)
C6	0.036 (2)	0.026 (2)	0.030 (2)	0.0017 (17)	−0.0005 (17)	0.0018 (17)
O2	0.063 (2)	0.054 (2)	0.056 (2)	0.0176 (18)	0.0296 (18)	0.0073 (17)
C11	0.043 (3)	0.038 (3)	0.043 (3)	0.010 (2)	−0.006 (2)	0.015 (2)
C1	0.044 (3)	0.031 (2)	0.033 (2)	0.0020 (19)	0.0056 (19)	−0.0015 (17)
C5	0.051 (3)	0.030 (2)	0.044 (3)	0.004 (2)	0.008 (2)	0.008 (2)
C12	0.062 (3)	0.031 (2)	0.051 (3)	0.004 (2)	−0.003 (2)	−0.003 (2)
C4	0.056 (3)	0.024 (2)	0.056 (3)	0.010 (2)	0.008 (2)	0.006 (2)
O1W	0.049 (2)	0.074 (3)	0.083 (3)	0.026 (2)	−0.0229 (19)	−0.046 (2)
N2	0.046 (2)	0.041 (2)	0.045 (2)	0.0095 (18)	−0.0008 (17)	0.0148 (18)
C13	0.066 (3)	0.045 (3)	0.037 (3)	0.013 (3)	0.007 (2)	−0.003 (2)
C10	0.049 (3)	0.060 (3)	0.063 (3)	0.010 (3)	−0.004 (2)	0.034 (3)
C9	0.068 (3)	0.031 (2)	0.040 (3)	0.005 (2)	−0.005 (2)	0.006 (2)
N3	0.173 (6)	0.051 (3)	0.049 (3)	0.057 (4)	−0.007 (3)	0.000 (2)
C8	0.155 (7)	0.043 (3)	0.039 (3)	0.042 (4)	−0.015 (3)	−0.001 (2)
O2W	0.235 (12)	0.026 (4)	0.017 (3)	0.038 (5)	0.034 (5)	0.010 (3)

### Geometric parameters (Å, °)

**Table:**

Cu1—N1	1.908 (3)	C11—C10	1.510 (7)
Cu1—N4	1.950 (3)	C5—C4	1.384 (7)
Cu1—O1	2.006 (3)	C5—H5	0.9300
Cu1—O3	2.048 (3)	C12—C13^i^	1.389 (7)
Cu1—O1W	2.217 (4)	C12—H12	0.9300
O3—C7	1.280 (5)	C4—H4	0.9300
O1—C1	1.278 (5)	O1W—H1WA	0.8301
N1—C2	1.322 (5)	O1W—H1WB	0.8599
N1—C6	1.339 (5)	N2—C9	1.319 (6)
C7—O4	1.218 (5)	N2—N3	1.338 (6)
C7—C6	1.517 (6)	N2—C10	1.466 (6)
N4—C9	1.302 (6)	C13—C12^i^	1.389 (7)
N4—C8	1.318 (7)	C13—H13	0.9300
C2—C3	1.377 (6)	C10—H10A	0.9700
C2—C1	1.520 (6)	C10—H10B	0.9700
C3—C4	1.382 (7)	C9—H9	0.9300
C3—H3	0.9300	N3—C8	1.313 (7)
C6—C5	1.367 (6)	C8—H8	0.9300
O2—C1	1.226 (5)	O2W—H2WA	0.8500
C11—C13	1.364 (8)	O2W—H2WB	0.8482
C11—C12	1.374 (7)		
			
N1—Cu1—N4	169.43 (16)	O2—C1—C2	118.8 (4)
N1—Cu1—O1	80.88 (13)	O1—C1—C2	114.4 (4)
N4—Cu1—O1	99.00 (14)	C6—C5—C4	118.7 (4)
N1—Cu1—O3	79.57 (13)	C6—C5—H5	120.6
N4—Cu1—O3	99.15 (13)	C4—C5—H5	120.6
O1—Cu1—O3	159.60 (14)	C11—C12—C13^i^	120.7 (5)
N1—Cu1—O1W	96.92 (16)	C11—C12—H12	119.7
N4—Cu1—O1W	93.53 (15)	C13^i^—C12—H12	119.7
O1—Cu1—O1W	99.18 (15)	C3—C4—C5	120.0 (4)
O3—Cu1—O1W	88.91 (16)	C3—C4—H4	120.0
C7—O3—Cu1	115.2 (3)	C5—C4—H4	120.0
C1—O1—Cu1	114.5 (3)	Cu1—O1W—H1WA	129.9
C2—N1—C6	122.9 (4)	Cu1—O1W—H1WB	112.7
C2—N1—Cu1	118.0 (3)	H1WA—O1W—H1WB	117.2
C6—N1—Cu1	119.1 (3)	C9—N2—N3	109.6 (4)
O4—C7—O3	125.5 (4)	C9—N2—C10	129.6 (5)
O4—C7—C6	120.6 (4)	N3—N2—C10	120.7 (4)
O3—C7—C6	113.9 (3)	C11—C13—C12^i^	120.6 (5)
C9—N4—C8	103.3 (4)	C11—C13—H13	119.7
C9—N4—Cu1	127.5 (3)	C12^i^—C13—H13	119.7
C8—N4—Cu1	129.2 (3)	N2—C10—C11	111.8 (4)
N1—C2—C3	119.7 (4)	N2—C10—H10A	109.2
N1—C2—C1	111.6 (4)	C11—C10—H10A	109.2
C3—C2—C1	128.7 (4)	N2—C10—H10B	109.2
C2—C3—C4	118.9 (4)	C11—C10—H10B	109.2
C2—C3—H3	120.6	H10A—C10—H10B	107.9
C4—C3—H3	120.6	N4—C9—N2	110.2 (4)
N1—C6—C5	119.9 (4)	N4—C9—H9	124.9
N1—C6—C7	111.7 (3)	N2—C9—H9	124.9
C5—C6—C7	128.5 (4)	C8—N3—N2	102.0 (5)
C13—C11—C12	118.7 (5)	N3—C8—N4	114.9 (5)
C13—C11—C10	120.4 (5)	N3—C8—H8	122.5
C12—C11—C10	120.9 (5)	N4—C8—H8	122.5
O2—C1—O1	126.7 (4)	H2WA—O2W—H2WB	137.0

Symmetry codes: (i) −*x*, −*y*+3, −*z*.

### Hydrogen-bond geometry (Å, °)

**Table:**

*D*—H···*A*	*D*—H	H···*A*	*D*···*A*	*D*—H···*A*
O2W—H2WA···O2W^ii^	0.85	2.15	2.920 (5)	151.
O2W—H2WB···O2W^iii^	0.85	1.96	2.807 (5)	179.
O1W—H1WA···O4^iv^	0.83	1.93	2.746 (5)	168.
O1W—H1WB···O3^v^	0.86	1.86	2.692 (5)	164.

Symmetry codes: (ii) −*x*+2, −*y*+2, −*z*; (iii) −*x*+1, −*y*+2, −*z*; (iv) −*x*, *y*+1/2, −*z*+1/2; (v) *x*+1, *y*, *z*.
